# Characterizing the anti-inflammatory and tissue protective actions of a novel Annexin A1 peptide

**DOI:** 10.1371/journal.pone.0175786

**Published:** 2017-04-13

**Authors:** Mauro Perretti, Clara Di Filippo, Michele D’Amico, Jesmond Dalli

**Affiliations:** 1 The William Harvey Research Institute, Barts and The London School of Medicine, Queen Mary University of London, Charterhouse Square, London, United Kingdom; 2 Department of Experimental Medicine, Second University of Naples, Naples, Italy; University of Calgary, CANADA

## Abstract

Inflammation in now appreciated to be at the centre of may diseases that affect Western civilization. Current therapeutics for managing these conditions may interfere with the host response leading to immune suppression. We recently developed an annexin (Anx) A1-derived peptide, coined CR-AnxA1_2-50_, which displays potent pro-resolving and tissue protective actions. Herein, we designed a novel peptide using CR-AnxA1_2-50_ as a template that was significantly more resistant to neutrophil-mediated degradation. This peptide, termed CR-AnxA1_2-48_, retained high affinity and specificity to the pro-resolving Lipoxin A_4_ receptor (ALX) with an IC_50_ of ~20nM. CR-AnxA1_2-48_ dose dependently (100fM-10nM) promoted the efferocytosis of apoptotic neutrophils, an action that was mediated by the murine orthologue of human ALX. The neutrophil-directed actions were also retained with human primary cells were CR-AnxA1_2-48_ reduced human neutrophil recruitment to activated endothelial cells at concentrations as low as 100 pM. This protective action was mediated by human ALX, since incubation of neutrophils with an anti-ALX antibody reversed this anti-inflammatory actions of CR-AnxA1_2-48_. Administration of this peptide to mice during dermal inflammation led to a significant and dose dependent decrease in neutrophil recruitment. This reduction in neutrophil numbers was more pronounced than that displayed by the parent peptide CR-AnxA1_2-50_. CR-AnxA1_2-48_ was also cardioprotecitve reducing infarct size and systemic chemokine (C-C motif) ligand 5 concentration following ischemia reperfusion injury. These findings identify CR-AnxA1_2-48_ as a new ALX agonist that regulates phagocyte responses and displays tissue-protective actions.

## Introduction

Inflammation is intrinsically host protective [1]. Recent evidence suggests that when resolution mechanisms become dysregulated the inflammatory response may be perpetuated leading to unabated inflammation and tissue destruction [2, 3]. A failed resolution response is now thought to be at least contributory to the onset and propagation of many inflammatory conditions afflicting western civilization including cardiovascular disease [4] and rheumatoid arthritis [5]. Therapeutics employed in the clinic to date to treat these inflammatory conditions aim to inhibit various mediators that promote the immune response. While this approach is effective at limiting inflammation in some instances it also carries severe side effects including immunosuppression with an elevated risk of infections [6, 7].

It is now well appreciated that in self-limited inflammation, i.e. when inflammation does not progress to chronicity, the body engages mechanisms that actively downregulate the production of inflammatory mediators as well as the clearance of leukocytes and cellular debris from the site of inflammation [8, 9]. In this context several families of molecules including specialized pro-resolving mediators [9], gaseous mediators (e.g. carbon monoxide [10] and hydrogen sulphide [11]) and proteins were recently described to regulate various aspects of the inflammation-resolution process. Amongst the proteins known to be central to regulating the termination of inflammation is Annexin A1 (AnxA1), a 37 KDa glucocorticoid-regulated protein. This protein regulates leukocyte trafficking in both murine [12] and human systems [13, 14], it also promotes the uptake and clearance of apoptotic cells by macrophages [15], a hallmark of resolution, and is organ protective[12]. The biological actions of this pro-resolving molecule are mediated by the Lipoxin A_4_ receptor (ALX) [8]. Mapping of the pharmacophore of this protein to the N-terminal portion lead to the development of peptides that replicate some of the biological actions of the parent protein [16]. The most widely studied AnxA1-derived peptide is a peptide based on the first 26 amino acid sequence, which displays similar bioactions to the parent protein, however does not retain the same receptor specificity as AnxA1. Indeed, the actions of this peptide are mediated by both the ALX receptor and the related formyl peptide receptor (FPR)1 [16]. Furthermore, this peptide displays significantly lower potency then the parent protein [16].

In recent studies, we developed a novel peptide modelled on the first 50 amino acids in the N-terminal portion of AnxA1 [17]. This peptide, coined, CR-AnxA1_2-50_, binds and activates the AnxA1 cognate receptor with a high degree of selectivity and specificity. It also retained the anti-inflammatory and pro-resolving actions of the parent protein, regulating neutrophil recruitment to the site of sterile inflammation in mice and neutrophil endothelial interactions with primary human cells. CR-AnxA1_2-50_ also accelerated the resolution of ongoing inflammation and promoted the uptake of apoptotic cells by macrophages, these being key pro-resolving actions [17].

Neutrophils play an important role in inactivating AnxA1 [12] and AnxA1-derived peptides [17] *via* proteinase mediate degradation [12]. Therefore in the present study we sought to enhance the potential therapeutic profile of CR-AnxA1_2-50_ by removing a recognition motif identified in previous studies to be important for neutrophil mediated degradation[17]. The resulting peptide displayed a high affinity to the ALX receptor and regulated neutrophil recruitment to the site of inflammation to a greater extent then CR-AnxA1_2-50_. The novel peptide also displayed potent cardioprotecive actions in murine cardiac reperfusion injury.

## Materials and methods

### Ethics

All animal studies were conducted with ethical approval from the Queen Mary University of London Local Ethical Review Committee and were conducted in accordance with the UK Home Office regulations (Guidance on the Operation of Animals, Scientific Procedures Act, 1986) and the Animal Care Ethical Committee of the 2^nd^ University of Naples. Human cells were prepared according to a protocol approved by the East London & The City Local Research Ethics Committee (Ref. 05/Q0603/34 ELCHA, London, United Kingdom).

### Cloning and expression of CR-ANXA1_2-50_ and CR-AnxA1_2-48_

Glycine-extended CR-ANXA1_2-50_ and CR-AnxA1_2-48_ were designed for cloning and expression using vectors for extracellular expression in *E*. *coli* as detailed in [17]. Briefly, the designed genes were synthesized by DNA 2.0 (Menlo Park, CA) using their codon optimization algorithm, followed by single amino acid modifications using PCR in the noted constructs. The digenic plasmid construct of each analog was used to transform the *E*. *coli* host strain, BLM6L, resulting in expression strains for each analog gene construct. These recombinant cell lines were screened for kanamycin resistance and growth at 37°C in a semi-defined inoculation media, as described in [18]; the plasmid constructs were confirmed with diagnostic restriction enzyme mapping, and final isolates were screened in shake flask experiments for the extracellular production of the peptide of interest, using a anion exchange-high pressure liquid chromatography. Selected isolates were further evaluated in bench scale fermentations. Each fermentation was run as a substrate limited, fed batch run with the induction of recombinant protein achieved using the chemical inducer, IPTG, which was incorporated into the feed. The fermentation was run under standard conditions of 32°C, pH 6.6, and dissolved oxygen at 80% by supplementation with O_2_ in media as described [18]. These fermentations were assayed and in some cases harvested between 23 and 31 hours post induction. For purification the fermentation was acidified and chilled; the conditioned media was harvested by centrifugation for downstream processing.

### Reverse phase-high pressure liquid chromatography

Purity of the AnxA1 peptides was determined using reverse phase-high pressure liquid chromatography isolation. Chromatography was carried out on a Thermo Electron BDS Hypersil C18 column (Thermo Fisher Scientific), 4.6 X 250 mm, 5 mm, 120 Å equilibrated with 0.1% TFA, 18% MeCN. Separation was achieved using a linear gradient from 20% B to 70% B (mobile phase A: 0.1% TFA; mobile phase B: 0.08% TFA, 90% MeCN) over 20 minutes. The column was operated at ambient temperature at a flow rate of 1.2 mL/min. The UV absorbance of the column effluent was monitored at 220 nm.

### Peptide-receptor interactions

Binding experiments were conducted as in [13]. Briefly FPR1-human embryonic kidney (HEK)–or ALX-HEK—transfected cells (1x10^6^ cells/ml) were incubated with increasing concentrations of cold CR-AnxA1_2-50_, CR-AnxA1_2-48_ (0.1nM-10μM) or PBS and a fixed concentration (50nM) of [^125^I-Tyr]-Ac2-26 for (1h, 4°C). Bound and unbound tracer were separated by filtration through Whatman GF/C glass microfibre filters (Kent, United Kingdom) using a vacuum manifold, and the amount of tracer bound to the cells was quantified by counting filters in a gamma-counter.

Receptor activation was monitored using the β-Arrestin PathHunter system (Discoverx) with experiments conducted as in [17] using HEK cells stably overexpressing recombinant human ALX receptors tagged with a prolink label of β-galactosidase (β-gal) and β-arrestin linked to the enzyme acceptor fragment of β-gal. In brief, cells were plated in 96-well plates (20,000 cells/well) 24 h before initiating experiments. CR-ANXA1_2-50_ and CR-AnxA1_2-48_ were incubated with cells (60 min, 37°C), and receptor activation was determined by measuring chemiluminescence using the PathHunter detection kit (Discoverx).

### Phagocytosis assay

Phagocytosis assay was conducted as previously described [17]. Briefly mice were injected with 1ml of a 2% BioGel (BioRad Laboratories, Hemel Hempstead, UK) solution i.p., 4 days later the peritoneum was lavaged using PBS + 3% EDTA. Cells were then passed through a 70-μm strainer to remove any remaining BioGel, washed twice with PBS, plated in a 24 well plate at 0.5x10^6^ cells per well and incubated overnight. Macrophages were incubated with CR-AnxA1_2-50,_ CR-AnxA1_2-50_ (10nM, 100pM or 100fM) or vehicle for 15 min. Then, 3x10^6^ fluorescently labeled apoptotic neutrophils were added per well and cells incubated for further 60 min at 37°C. The extracellular fluorescence was quenched using Trypan Blue and extent of phagocytosis assessed using a NOVOstar microplate reader (BMG LABTECH Ltd., Aylesbury).

### Murine airpouch

Dorsal subcutaneous air-pouches were prepared in male CD1 mice (6 to 8 weeks of age; Charles River) as described in [19]. PBS (PBS+0.01% DMSO), CR-AnxA1_2-50_ (5pg-5μg/mouse) or CR-AnxA1_2-48_ were administered (5pg-5μg/mouse) i.v. 10 min prior to intra-pouch administration of IL-1β (10ng suspended in 500 μl of 0.5% carboxymethyl-cellulose; BDH, Dorset, UK). After 4h lavages were collected and the number of extravasated neutrophils assessed by light microscopy and flow cytometry as described in [20]. The doses and time intervals were selected based on published findings with CR-AnxA1_2-50_ and related native peptide [17].

### *In vitro* flow chamber

Human peripheral blood neutrophils and human umbilical endothelial veins (HUVEC) were isolated and flow chamber experiments were conducted as in [17]. Briefly HUVEC were plated in 35mm dish until confluent and then incubated with TNF-α (10 ng/ml, 4h; R&D systems). Neutrophils were suspended at 1x10^6^/ml in Dulbecco’s PBS and incubated with CR-AnxA1_2-50_, CR-AnxA1_2-48_ (0.1pM-10nM) or PBS (PBS+0.01% DMSO) for 10min, 37°C. These were then perfused over the HUVEC monolayers (8 min, 1 dyne/cm^2^), and six fields were recorded for 10 second intervals. Total number of interacting neutrophils was assessed as in [13]. In designated experiments neutrophils were incubated for 10 min with anti-ALX mAb (10μg/ml, clone FN-1D6-A1, Genovac, Freiburg, Germany), or isotype controls, mouse IgG1 (BD biosciences) prior to incubation with PBS, CR-AnxA1_2-50_ (100pM) or CR-AnxA1_2-48_ (100pM) and perfused over activated endothelial cells.

### Murine ischemia reperfusion

Murine myocardial ischemia was induced as in [21]. After a 25 min period of myocardial ischemia by closing the left anterior descending coronary artery (LADCA), the clip was removed so that the tension on the ligature was released and reperfusion occurred for 2 h. PBS (PBS+0.01%DMSO), CR-AnxA1_2-50_ (5μg/mouse) or CR-AnxA1_2-48_ (5μg/mouse) were administered via i.v. injection and blood was collected 2h post reperfusion. The doses and time intervals were selected based on published findings with CR-AnxA1_2-50_ and related native peptide [17]. Plasma CCL5 (R&D System, Abingdon, UK) was measured using ELISA and following manufacturer’s instructions. Two hours later, the LADCA was then re-occluded and mice injected with Evans blue dye (1ml, 2% wv^-1^) staining the perfused and viable myocardium, leaving the occluded and necrotic vascular bed uncolored. The heart was removed the left ventricle (LV) excised and weighed. This was then sectioned and the area at risk (AR) was separated from the non-ischaemic myocardium. To distinguish between ischemic and infarcted tissue, the AR was cut into small pieces and incubated with *p*-nitro-blue tetrazolium (NBT, 0.5 mgml^-1^, 20 min, 37°C).

### Statistics

Results are presented as mean ± SEM. Statistical analysis was performed using the paired, unpaired Student's t test, or one-way ANOVA followed by a Dunnett’s post hoc test as appropriate. In all instances P<0.05 was considered to be significant.

## Results

### CR-AnxA1_2-48_ displays elevated resistance to neutrophil mediated degradation and binds ALX with high affinity

**Fig 1:**
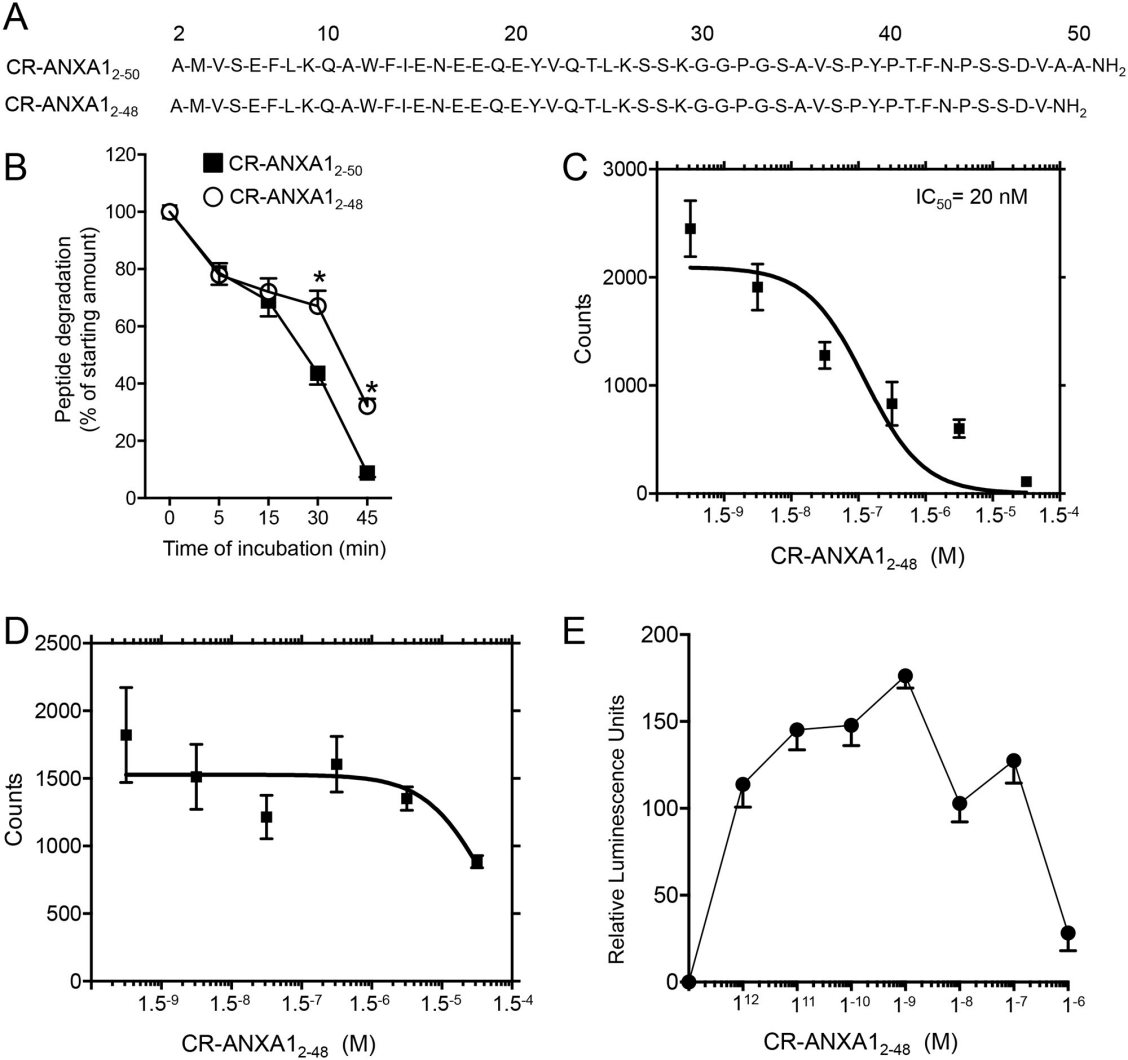
CR-AnxA1_2-48_ is resistant to neutrophil mediated cleavage and selectively binds the ALX receptor. **(A)** Aminoacid sequences for CR-AnxA1_2–50_ and CR-AnxA1_2–48_ **(B)** Plots depicting the amount of peptide remaining at the indicated intervals in incubations of human neutrophil with CR-AnxA1_2–50_ or CR-AnxA1_2–48._ Results are mean ± SEM of n = 4 distinct incubations with different neutrophil preparations. *p < 0.05 versus AnxA1_2–48_ incubations. **(C,D)** Binding affinity of CR-AnxA1_2–48_ to (C) human ALX-overexpressing and (D) FPR1-overexpressing HEK cells was assessed in a competitive binding assay using [^125^I]–Ac2-26. (**E**) Agonist activity of CR-AnxA1_2–48_ was investigated using β-arrestin stably expressing FPR2/ALX. Results for C-E are mean ± SEM of 3–4 independent experiments.

Results from published studies indicated that in addition to Valine_25_; Alanine_49_ and Alanine_50_ were also found to be important recognition sites for neutrophil protease enzymes [17]. Therefore, we designed a peptide that in addition to having a substitution of Valine_25_ to Leucine, which we previously found to confer resistance to proteinease-3 and neutrophil elastae [17], also lacked Alanines at positions 49 and 50 (Fig 1A). Incubation of this peptide, coined CR-AnxA1_2-48_, with activated human neutrophils demonstrated that removal of these two amino acids conferred a significant increase in resistance to neutrophil mediated cleavage when compared to its parent peptide CR-AnxA1_2-50_ (Fig 1B).

We next tested whether the modifications influenced its affinity to the ALX receptor. For this purpose we incubated HEK cells overexpressing the human ALX receptor and tested the ability of CR-AnxA1_2-48_ to competitively displace a radiolabelled tracer [13]. Incubation CR-AnxA1_2-48_ with I^125^ labelled Ac2-26 peptide [22] demonstrated that CR-AnxA1_2-48_ displayed a high affinity to the ALX receptor with an IC_50_ of 20nM (Fig 1C). Of note, incubation of CR-AnxA1_2-48_ with FPR1-HEK cells did not produce specific competition curves in the presence of the same tracer (Fig 1D). We next tested whether this peptide also activated the ALX receptor. For this purpose we utilized the β-Arrestin PathHunter system where ligand binding leads to a luminescent signal. Incubation of ALX-overexpressing HEK cells with CR-AnxA1_2-48_ lead to a dose dependent increase in luminescent signal that displayed a characteristic bell shape does response (Fig 1E). Together these results demonstrate that CR-AnxA1_2-48_ displays higher resistance to neutrophil degradation, while retaining a high degree of affinity and specificity to the pro-resolving receptor ALX.

### Pro-resolving and anti-inflammatory actions of CR-AnxA1_2-48_ are mediated by ALX

**Fig 2:**
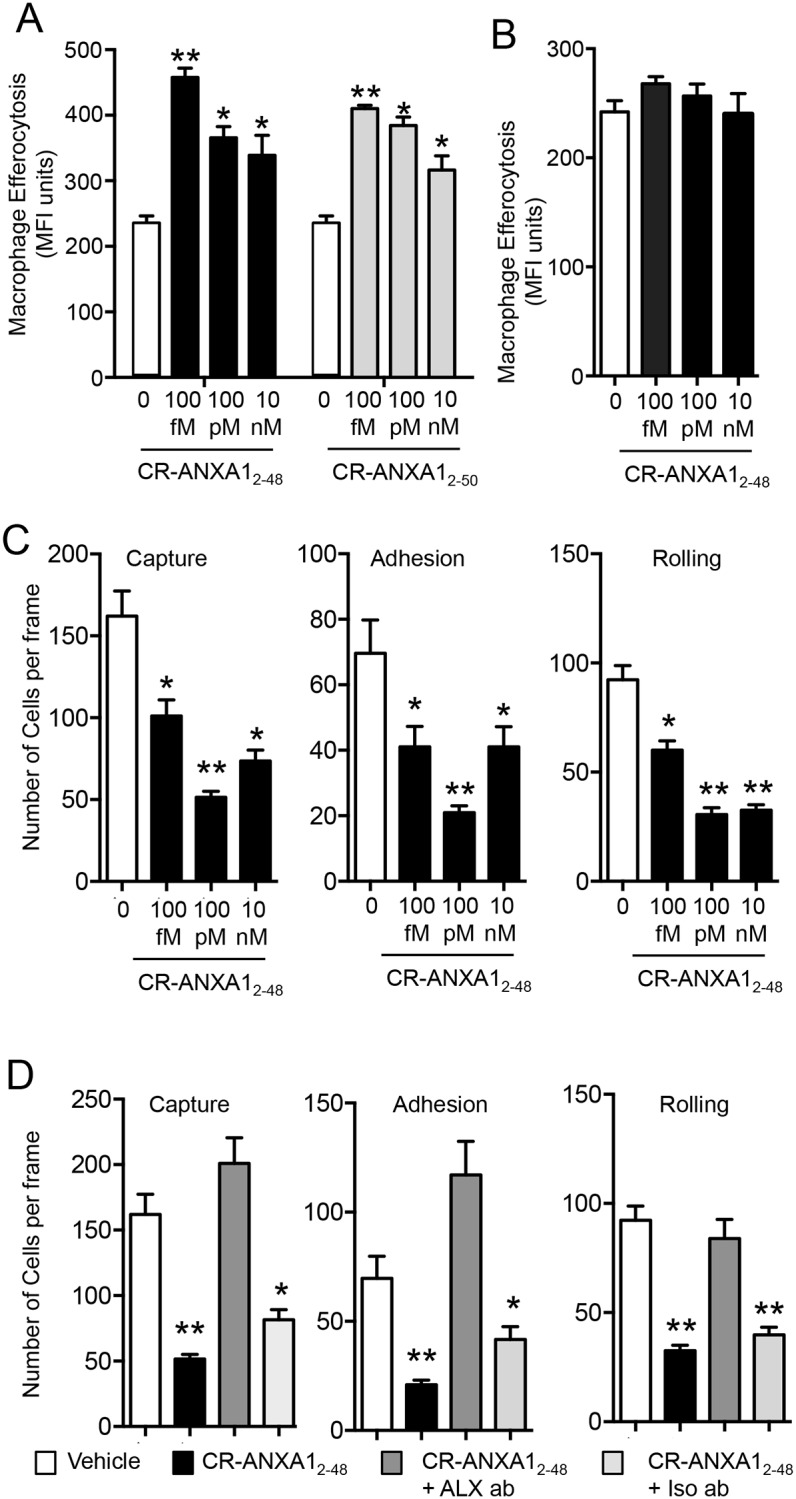
Anti-inflammatory and pro-resolving actions of CR-AnxA1_2-48_ with mouse and human leukocytes. **(A)** Plots depicting phagocytosis of apoptotic human neutrophils (efferocytosis) by macrophages incubated with or without the indicated concentrations of CR-AnxA1_2–50_ and CR-AnxA1_2–48_. (**B**) Bar graphs depicting phagocytosis of apoptotic human neutrophils (efferocytosis) by bone marrow derived macrophages from Alx/Fp2/3^-/-^ mice incubated with or without the indicated concentrations of CR-AnxA1_2–48_. Results are mean ± SEM. n = 4 mice per group per time point; *p < 0.05, **p < 0.01, versus vehicle group. (**C**) Plots depicting the number of human neutrophils interacting with activated human endothelial cells incubated with or without the indicated concentrations of CR-AnxA1_2–48_.. (**D**) Bar graphs illustrating the number of neutrophils interacting with activated endothelial cells. Results for C,D are mean ± SEM of n = 4 independent cellular preparations *p < 0.05 versus PBS, **p < 0.01 versus vehicle control.

Having found that CR-AnxA1_2-48_ activated ALX we next tested whether it carried anti-inflammatory and pro-resolving actions through this molecular target. We first assessed its ability to promote efferocytosis of apoptotic neutrophils a key step in the resolution of inflammation [3]. Incubation of murine macrophages with CR-AnxA1_2-48_ led to a dose dependent increase in macrophage efferocytosis (40–80%; Fig 2A). The activity of this peptide at promoting efferocytosis was also found to be comparable to that displayed by its parent peptide CR-AnxA1_2-50_ (Fig 2A). We next tested whether the murine orthologue of the ALX receptor was responsible for mediating this pro-resolving action of CR-AnxA1_2-48_. Incubation of cells lacking the ALX receptor with CR-AnxA1_2-48_ did not lead to a significant increase in macrophage efferocytosis when compared with cells incubated with PBS alone (Fig 2B).

Having assessed the ability of the new AnxA1-derived peptide in regulating macrophage response, we next investigated whether the actions of CR-AnxA1_2-50_ on neutrophil reactivity [17] were retained with CR-AnxA1_2-48_. Incubation of this peptide with isolated human neutrophils gave concentration-dependent decreases in neutrophil interactions to activated primary endothelial cells. This was reflected in significant decreases in both the numbers of adherent as well as rolling cells on the endothelial monolayer (Fig 2C). Incubation of human neutrophils with a neutralizing antibody to the ALX receptor reversed this biological action, reverting the number of cells interacting with the activated vascular endothelial cells back to levels observed in vehicle control cells (Fig 2D). These actions were also specific to the ALX receptor since incubation of neutrophils with an isotype control antibody did not reverse the biological actions of CR-AnxA1_2-48_ (Fig 2D). Together these findings demonstrate that CR-AnxA1_2-48_ displays potent leukocyte directed anti-inflammatory and pro-resolving actions that are mediated by the receptor ALX.

### CR-AnxA1_2-48_ potently regulates neutrophil recruitment in vivo

**Fig 3:**
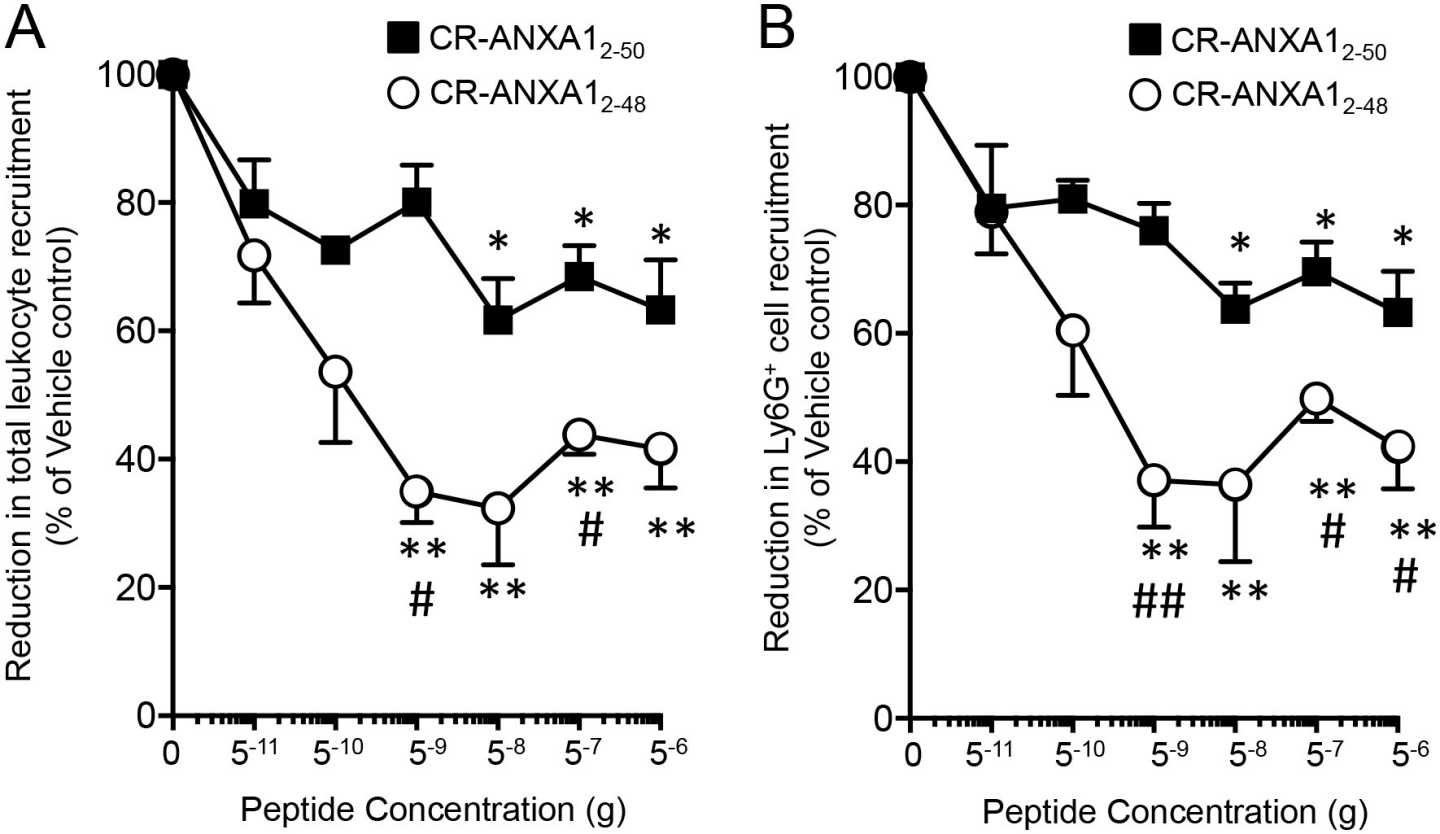
CR-AnxA1_2–48_ regulated neutrophil recruitment *in vivo*. Graphs illustrate (A) total leukocyte numbers and (B) the number of Ly6G^+^ cells quantified using light microscopy and flow cytometry (see Materials and Methods for details). Results are mean ± SEM of n = 4 mice per group; *p < 0.05, **p < 0.01, versus vehicle group.

Since CR-AnxA1_2-48_ displayed potent biological actions with isolated leukocytes we next tested its ability to regulate leukocyte responses *in vivo*. For this purpose we used the IL-1β initiated air-pouch model of dermal inflammation. Administration of IL-1β to mice gave a robust leukocyte influx, with neutrophils being the predominant cell type. Administration of CR-AnxA1_2-48_ immediately prior to the onset of inflammation gave dose-dependent decreases in both the total number of leukocytes and the number of neutrophils, identified as Ly6G^+^ cells, recruited into the pouches (Fig 3). This reduction in neutrophil recruitment was displayed with doses as low as 5ng/mouse where we found > 60% decrease in the number of neutrophils recruited to the site of inflammation. In these experimental settings, CR-AnxA1_2-48_ was more potent then the parent peptide CR-AnxA1_2-50_. Thus, these results demonstrate that CR-AnxA1_2-48_ carries leukocyte directed actions *in vivo*, with higher potency than CR-AnxA1_2-50_.

### CR-AnxA1_2-48_ is cardioprotective during ischemia-reperfusion mediated tissue injury

**Fig 4:**
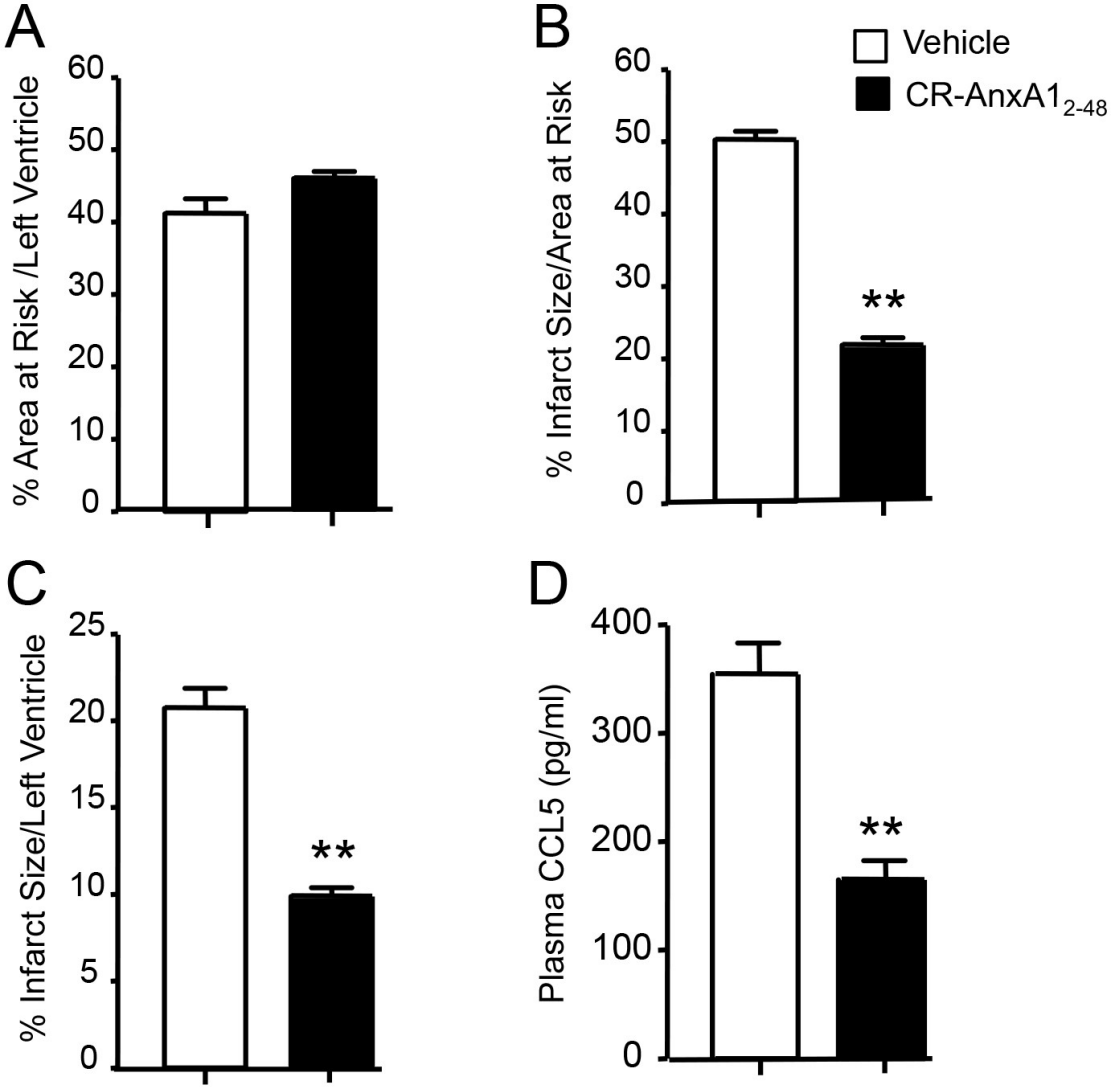
CR-AnxA1_2–48_ displays cardioprotective actions in murine ischemia reperfusion mediated injury. Graphs depict (**A**) Relative area at risk to total ventricle size in each group (**B-C**) Quantitative analyses for tissue damage expressed as (B) infarct size/area at risk and (C) infarct size/left ventricle. (D) Plasma CCL5 concentrations. Results are mean ± SEM of n = 6 mice per group. **p < 0.01 versus Vehicle group.

Next we tested whether CR-AnxA1_2-48_ also displayed tissue protective actions during cardiac-ischemia reperfusion where leukocyte migration and activation leads to tissue damage [23]. Ligation of the left descending coronary artery for 25 min and reperfusion for 120 min led to marked damage within the left ventricle where ~50% of the area at risk was compromised, corresponding to ~20% of the left ventricle (Fig 4A–4C). Administration of CR-AnxA1_2-48_ immediately prior to reperfusion gave significant protection, reducing the amount of damaged tissue by >50% when compared to vehicle control (Fig 4B and 4C). In addition, CR-AnxA1_2-48_ also reduced plasma levels of the pro-inflammatory cytokine chemokine (C-C motif) ligand 5 or CCL5 (Fig 4D).

## Discussion

In the present study we characterized the biological actions of a novel AnxA1-derived peptide. Results from these experiments demonstrate that CR-AnxA1_2–48_ displays increased stability to neutrophil mediated degradation when compared to its parent peptide (Fig 1). This peptide also displayed a high affinity to the pro-resolving receptor ALX *via* which it exerted its potent anti-inflammatory and pro-resolving actions on phagocytes (Figs 1 and 2). Administration of CR-AnxA1_2–48_ to experimental animals, demonstrated that the biological actions identified *in vitro* were retained *in vivo*, where this peptide regulated neutrophil trafficking and displayed tissue-protective properties during leukocyte mediated tissue damage (Figs 3 and 4).

Recent studies suggest that utilizing therapeutics which harness processes within the resolution of acute inflammation rather then inhibiting inflammation altogether may yield therapeutics that are devoid of the unwanted side effects associated with current medicines [4, 5, 24]. AnxA1 is a pro-resolving protein that regulates immune response to promote the termination of both acute and chronic inflammation [25]. Studies assessing the activity of this protein indicate that AnxA1 loses its protective actions during ongoing inflammation [17, 19, 26]. In this aspect several mechanisms are now appreciated to lead to this reduced activity, these include the down regulation of its cognate receptor ALX, the decrease in expression of the protein and increased cleavage of the n-terminal portion, the pharmacophore of the protein [12, 27]. Given that AnxA1 reprograms the host immune response to terminate inflammation and in doing so does not interfere with the host’s ability to combat infections [28] recent studies have investigated the actions of peptides derived from the N-terminal portion of this protein as potential therapeutic leads [17, 29–31]. In this context, peptides spanning the first 50 amino acids of the AnxA1 N-terminal regulate both murine and human leukocyte responses activating resolution programs [17]. Assessment of the cleavage patterns by neutrophil proteinase enzymes identified several recognition sites for proteinase-mediated degradation of these peptides [17]. Thus, in the present study we designed a new peptide that displayed enhanced resistance to neutrophil-mediated degradation by modifying two of the recognition sites. The first was the substitution Valine_25_ to Leucine that we previously found to increase stability while not altering function of the peptide and the second was to remove Alanine_49_ and Alanine_50_ (Fig 1) and [17]. These modifications yielded a peptide that retained the leukocyte directed actions of the natural sequence (Figs 2,3 and 4 and [17]) with enhanced stability towards neutrophil mediated degradation when compared to both the natural sequence [17] and the cleavage resistant peptide we previously designed (Fig 1).

The ALX receptor is a G-protein coupled receptor that mediates the biological actions of a number of pro-resolving molecules including the essential fatty acid derived specialized pro-resolving mediators Resolvin D1, Resolvin D3 and Lipoxin A_4_ [32]. Of note, this receptor also mediates some of the biological actions of the acute phase protein Serum Amyloid A and is suggested to recognize endogenous formlyated peptides, although the binding affinity to the formylated peptide fMLF to ALX is about a log order lower then that to FPR1 [33]. Recent studies demonstrate that only pro-resolving molecules, including AnxA1, are able to promote the homodimerization of the ALX receptor, a process that leads to the activation of a host protective signalling cascade involving a p38/MAPK-activated protein kinase/heat shock protein 27 signalling signature [27]. In the present studies we found CR-AnxA1_2–48_ retained a high affinity to ALX with an IC_50_ of 20nM and displayed a characteristic bell-shaped dose response in its ability to activate this receptor (Fig 1). This bell shape response is also reflected in its ability to regulate neutrophil responses *in vitro* and *in vivo*, an action that is in line with that protective properties of AnxA1 [12] as well as other pro-resolving agonists to this receptor [34].

In summation, results from the present experiments provide evidence for the potent biological actions of CR-AnxA1_2–48_, a peptide derived from the pharmacophore of the pro-resolving and tissue protective protein AnxA1. This peptide in addition to displaying enhanced stability to human neutrophil-mediated cleavage, also exerts potent (fM) leukocyte directed actions regulating host responses during acute inflammation and protects the heart from leukocyte mediated tissue damage. Thus, taken together these findings characterize a novel pro-resolution agonist to the ALX receptor that may be used to treat ongoing inflammation without interfering with the host immune response.
